# Functional, physiological and subjective responses to concurrent neuromuscular electrical stimulation (NMES) exercise in adult cancer survivors: a controlled prospective study

**DOI:** 10.1038/s41598-020-71006-w

**Published:** 2020-08-19

**Authors:** Dominic O’Connor, Olive Lennon, Matilde Mora Fernandez, Gabriel Ruiz Signorelli, Brian Caulfield

**Affiliations:** 1 School of Public Health, Physiotherapy and Sports Science, University College Dublin, Dublin, Ireland; 2 The Insight Centre for Data Analytics, O’Brien Centre for Science, University College Dublin, Belfield Campus, Dublin, Ireland; 3 Clinica Oncoavanze, Seville, Spain; 4 University of Seville, Seville, Spain

**Keywords:** Cancer, Physiology, Ageing, Quality of life, Outcomes research

## Abstract

The primary aim of this study was to investigate the functional, physiological and subjective responses to NMES exercise in cancer patients. Participants with a cancer diagnosis, currently undergoing treatment, and an had an Eastern Cooperative Oncology Group (ECOG) performance status (ECOG) of 1 and 2 were recommended to participate by their oncologist. Following a 2-week, no-NMES control period, each participant was asked to undertake a concurrent NMES exercise intervention over a 4-week period. Functional muscle strength [30 s sit-to-stand (30STS)], mobility [timed up and go (TUG)], exercise capacity [6-min walk test (6MWT)] and health related quality of life (HR-QoL) were assessed at baseline 1 (BL1), 2-week post control (BL2) and post 4-week NMES exercise intervention (POST). Physiological and subjective responses to LF-NMES were assessed during a 10-stage incremental session, recorded at BL2 and POST. Fourteen participants [mean age: 62 years (10)] completed the intervention. No adverse events were reported. 30STS (+ 2.4 reps, *p* = .007), and 6MWT (+ 44.3 m, *p* = .028) significantly improved after the intervention. No changes in TUG or HR-QoL were observed at POST. Concurrent NMES exercise may be an effective exercise intervention for augmenting physical function in participants with cancer and moderate and poor functional status. Implications for cancer survivors: By allowing participants to achieve therapeutic levels of exercise, concurrent NMES may be an effective supportive intervention in cancer rehabilitation.

## Introduction

Both cancer pathology and associated life extending treatments are linked to complications which can impair physical function and quality of life. Some complications are acute in nature, persisting across a treatment cycle and normalising during treatment recovery. However, common chronic complications following cancer treatment can persist for years. For example, fatigue, which is seen in almost 100% of patients, may persist for up to 10-years post treatment cessation^1,2^. Although poorly understood, the mechanisms behind cancer related fatigue have been linked to the role of skeletal muscle^3^. Common treatments such as chemotherapy and hormone therapy can have catastrophic effects on skeletal muscle structure and function, with an accelerated aging phenotype observed (muscle loss of 3.9% over 100 days of chemotherapy vs 1% per year during normal ageing)^4^. These losses are associated with impaired muscle strength in common cancers (~ 25% lower than healthy counterparts)^5,6^. Chemotherapy can also impair peak oxygen capacity (VO_2peak_), leading to poorer exercise tolerance^7^. These treatment complications can increase sedentary behaviour, impair physical function and compromise independence.

Conventional exercise (i.e. aerobic and resistance exercise) is now recognised as a primary adjunct therapy to help offset disease/treatment complications. There is strong evidence to support prescription of conventional exercise to improve cancer related health outcomes including fatigue, quality of life, and physical function across the cancer trajectory^8^. However, despite its recognised benefits, muscle strengthening, and aerobic exercise can be challenging in this population. Many individuals with impaired functional status while undergoing treatment experience exercise limiting symptoms. This translates to as few as 5% of individuals who are undergoing treatment being physically active^9^. Therefore, alternative exercise methods which can be undertaken during cancer treatment to minimise the loss of or augment functional outcomes and accelerate return to conventional exercise are warranted.

Neuromuscular electrical stimulation (NMES) is an emerging field in oncology rehabilitation^10^. High frequency tetanic NMES (HF-NMES) has a proven muscle strengthening effect using frequencies ≥ 20 Hz in healthy and patient populations^11^. Emerging evidence suggests that the application of sub-tetanic low frequency NMES (4 Hz, LF-NMES) can elicit a comfortable and sustainable aerobic exercise response, and enhance aerobic exercise capacity and exercise endurance in healthy and patient populations^12,13^. Recently, a concurrent model of NMES exercise delivery (both LF and HF-NMES phases) has been evaluated in patients with mixed cancer diagnoses^14,15^. This early work involving a case series and a case report has demonstrated safety, feasibility and patterns of improvement in functional and quality of life outcomes.

Preliminary, exploratory work in an oncology population suggests that concurrent NMES exercise may be best implemented in more deconditioned individuals to enhance functional outcomes^14,15^. This is a common finding in the general NMES literature^16–18^, and is a pragmatic consideration given the exercise limiting symptoms experienced by those with advanced disease stages. However, adherence to concurrent NMES exercise for extremely deconditioned individuals can be affected by the ability to apply NMES garments unsupervised^19^. This is unsurprising given that up to one third of those with cancer experience difficulty in basic activities of daily living including getting dressed^20^. Therefore, this may have important implications for long-term compliance to home-based unsupervised NMES exercise. In addition, no studies have investigated the functional response to concurrent NMES exercise in a controlled setting. Furthermore, although the physiological response to LF-NMES has been investigated in healthy participants^21^, the physiological and subjective response in an cancer population is currently unknown.

Therefore, the primary aim of this prospective controlled study was to assess the effects of concurrent NMES exercise in comparison to no NMES exercise intervention on functional and quality of life outcomes for participants undergoing active treatment who were unable to exercise independently in cancer rehabilitation. A secondary aim was to investigate the physiological response to LF-NMES was examined using a series of physiological and subjective dependent variables.

## Methodology

### Study participants

Participants were volunteers by self-selection who had a cancer diagnosis, were currently undergoing treatment (chemotherapy or hormone therapy) and had an ECOG performance status of 1 or 2. They were recommended to participate by their oncologist where physical limitations due to muscle weakness and asthenia which limited voluntary exercise participation were identified. Due to the subjective nature of the ECOG scale^22^, baseline 6-min walk test (6MWT) distance was also used to categorise participants according to functional status, (ECOG 1: > 350 m, and ECOG 2 < 350 m). 6-min walk test scores of < 350 m are classified as poor in comparator clinical populations^23^ and have been shown to correlate with performance status in patients with recurrent glioma^24^. Participants were excluded if they had limitations which affected their ability to complete functional exercise tests, a serious, uncontrolled cardiac arrhythmia, any cognitive impairment which may affect their ability to apply NMES safely without direct supervision, deep vein thrombosis within the last 6 months, metastatic lesions of the femur, or a cardiac pacemaker. Prior to study entry, participants provided written informed consent.

### Experimental design

This was a one group pre-test post-test prospective cohort study design comprising a 2-week control period (maintenance of habitual lifestyle, Wk 0–2) and a 4-week intervention period (Wk 3–6). Functional outcomes [30 s sit-to-stand (30STS), 6-min walk test (6MWT), timed up and go (TUG)], participant reported outcomes [(PRO’s), health related quality of life (HR-QoL)] and physical activity levels (PA) were recorded at three separate time points: start of control period [Baseline 1 (BL1)], post 2-week control period (BL2), and post 4-week NMES exercise intervention (POST). All participants were required to attend the university laboratory at each time point for assessment. The two-week control period was designed to show relative stability of the outcomes without intervention or possible decline as supported by the literature in this area^25^. In addition, this experimental design offset the need for a no/sham NMES control group and was selected due to practical reasons (high attrition rates associated with exercise interventions in cancer populations). To help control for cancer treatment effects, participants entered the study at least 1 week after commencing a treatment cycle, with testing sessions at BL2 and POST occurring at least 1 week before/after a treatment cycle.

**Figure 1:**
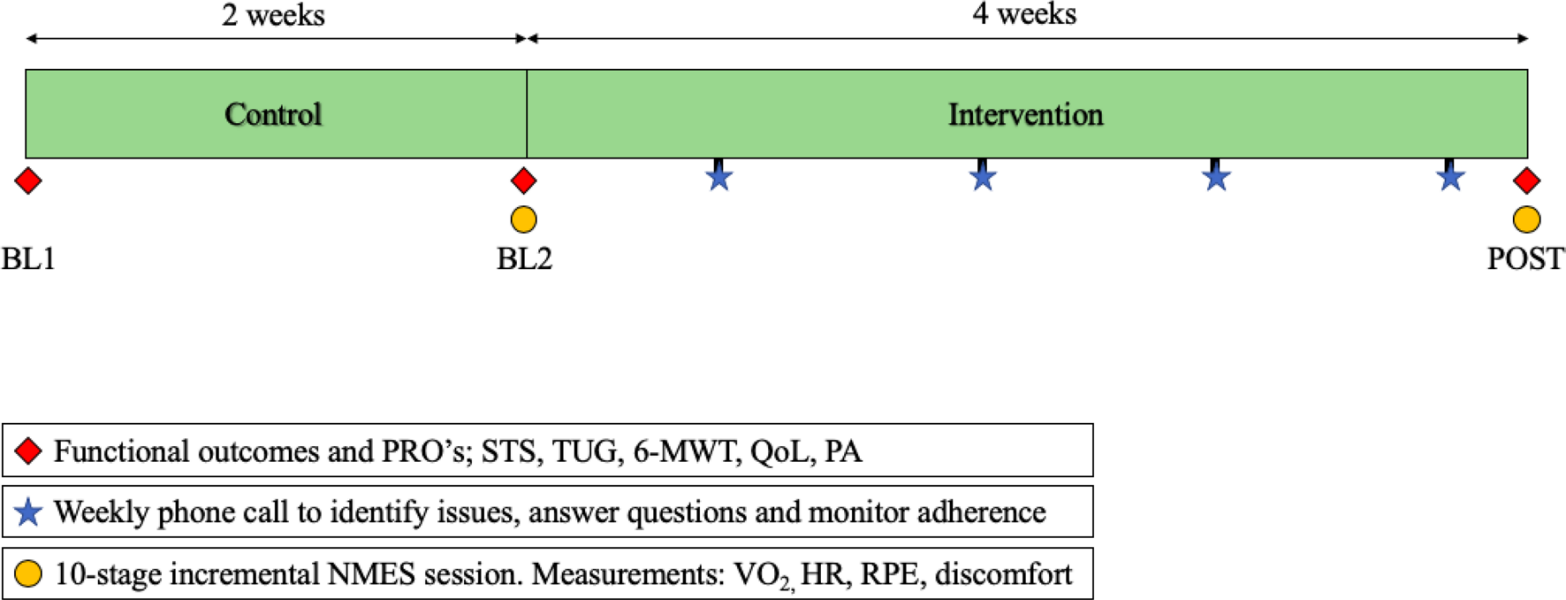
Schematic of study design.

Physiological [relative energy cost (VO_2_), heart rate (HR)] and subjective (rate of perceived exertion (RPE) and discomfort) outcome measures were recorded during two supervised 10-stage incremental NMES sessions to assess change in these outcomes selected as indices of improved cardiorespiratory fitness and intervention tolerability. These incremental sessions occurred at BL2 and POST. A schematic of the study design is depicted in Fig. 1.

### Primary outcome

#### Sit to stand performance

Lower limb functional muscle strength was assessed using the 30STS. The 30STS required patients to stand up from, and sit down on a 45 cm padded chair with no armrests as many times as possible in 30 s. Participants could use their hands to help them stand if required and were provided standardised verbal encouragement to continue to sit and stand throughout the test. Participants completed one trial. A change in 30STS score of ≥ 2 reps was considered the minimal clinically important difference (MCID) in line with the published literature^26,27^.

### Secondary outcomes

#### 6-min walk test (6MWT)

Functional exercise capacity was assessed using a 6MWT, a simple walking test that is often used as a surrogate measure of aerobic capacity. Participants were instructed to walk as far as possible in the 6-min, back and forth along a 20-m corridor, turning briskly around the markers at each end. Participants could slow down, stop and rest if necessary. Standardised moderate verbal encouragement was provided every 2 min to each patient by the same investigator. The distance walked in 6 min was recorded to the nearest meter. An improvement in distance of 30.5 m was considered the MCID, in line with the published literature^28^.

#### Timed up and go test (TUG)

Functional mobility was assessed using the TUG. Patients were required to stand up from a 45 cm chair, walk 3 m, turn around, walk 3 m back and sit down again, walking at their referred pace. The use of a walking aid was allowed. The test was completed twice, with the best score recorded. A change of 3 s in TUG time is considered the MCID for this measure^29^.

#### Health related quality of life (QoL)

The multidimensional European Organisation for the Research and Treatment of Cancer Quality of Life Questionnaire Core 30 (EORTC QLQ-C30) was used to assess HR-QoL. Using the EORTC scoring manual, a linear transformation was used to standardise the raw score, so that scores ranged from 0 to 100. A higher score represents a higher level of Global QoL and functioning. A change in functioning subscale and fatigue symptom scale score of at least 10 points was considered the MCID^30^.

#### Physical activity levels

Self-reported physical activity was measured using the International Physical Activity Questionnaire short form (IPAQ-sf). The IPAQ-sf is a valid and reliable^31^, seven item measure of four activity domains: vigorous intensity activity, moderate intensity activity, walking, and sitting. Participants report the frequency and duration of activity across each domain over the previous 7-days. Based on responses, participants can be categorised as low activity, moderate activity or high activity.

### Physiological and subjective responses to LF-NMES

#### Incremental NMES session

To assess the physiological and subjective response to LF-NMES, each participant completed a 10-stage incremental NMES protocol, on two separate occasions, at BL2 prior to beginning the intervention, and upon completion of the intervention POST. These sessions were carried out using a low pulse frequency (4 Hz, 620 μs, continuous) with the participant in the semi-fowler position. During the session, stimulation intensity was increased in equal increments (10%) every 3 min from a starting point of 14 mA (10% of maximum output: 140 mA) to reach initial maximum tolerable intensity. If participants, at the start of a new stage could not tolerate an increase of + 10%, the maximum tolerable increase was achieved prior to termination of the session at the end of that stage.

Expired gases (VO_2_) were measured breath-by-breath using an online gas analyser (Ultima CPX; MGC Diagnostics, Minneapolis, United States) and heart rate (HR) was recorded using wireless telemetry (Polar Electro, Kempele, Finland), at rest and throughout the incremental protocol. During the last 30-s of each stage, participants rated their perceived exertion and levels of discomfort. These variables were assessed using the RPE Borg’s 6–20 scale and an 11-point numerical rating scale (NRS) where participants were asked to rate their discomfort on a 0–10 scale where 0 indicates “no discomfort” and 10 indicates “worst possible discomfort/pain”. The maximum intensity achieved was used as the starting intensity for the LF-NMES phase of the home-based NMES exercise sessions.

Following the first incremental protocol, participants completed one supervised HF-NMES session (15 min), whereby they were encouraged to modulate stimulation intensity to establish their maximum tolerable intensity. The initial supervised incremental and HF-NMES session also acted as a familiarisation session whereby participants could ask questions regarding both LF and HF-NMES phases and were shown the correct and safe use of the NMES equipment by the study investigator. Participant also received written instructions which could be referred to at home.

### Protocol adherence

To further examine the effectiveness of the NMES exercise protocol, protocol adherence (no of sessions completed), the maximum current intensity achieved for LF-NMES and HF-NMES programmes, and the duration of NMES exercise completed across the intervention for both LF and HF-NMES phases were assessed at POST.

### NMES intervention

The NMES exercise intervention has previously been described in detail^14^. In brief, the four week concurrent NMES exercise intervention was delivered using a hand-held muscle stimulation unit (INKO RS, BioMedical Research Ltd, Galway, Ireland), and four adhesive gel electrodes (17 × 10.3 cm) placed on each leg (× 2 proximal and distal quadriceps, × 2 proximal and distal hamstrings) and applied via a pair of neoprene garments which were secured by velcro straps. The participants trained at home using a standard weekly progressive prescription (14 sessions) and were not supervised during NMES exercise. The prescription was personalised weekly following phone calls with each participant. The information gathered included subjective feedback and training diary information (sessions completed, stimulation intensity progression, session RPE) which informed session frequency and duration progression. The protocol delivered LF (13–45 min) and HF-NMES (15 min) phases during each session (total session time: 28–60 min).

As tolerability is a major determinant of the response to NMES^18^, a novel intermittent delivery of the LF-NMES programme was developed and was available for each patient in week 1. This method reduces the pulse width from 620 μs to 300 μs, used as a means of introducing relative ‘rest’ periods to the intermittent programme to accommodate habituation for unaccustomed users. However, participants could progress directly to continuous delivery, where deemed appropriate during the 10-stage incremental NMES protocol (could tolerate current intensities beyond 15-min, i.e. ≥ 70 mA).

Personalisation and progression in the LF-NMES session protocol involved increased weekly session duration (5–10 min per week). In the HF-NMES protocol, the duty cycle (on:off cycle) increased weekly from 2 s:15 s to 5 s:15 s to 5 s:10 s and constant thereafter as previously reported^14^. Session frequency progressed weekly from 2 × /week in week 1, to 5 × /week in week 4. However due to issues identified previously regarding increased sensitivity to NMES and high fatigue levels in the days following treatment infusion, a periodised approach was adopted to help maintain adherence. Participants were instructed to use the units on the day of infusion prior to treatment, and lower NMES exercise intensity in the 2–3 days immediately post treatment to sensory threshold, or motor threshold if tolerable. Participants were instructed to return to pre-treatment intensities 3–5 days post treatment when symptoms may have subsided. This strategy was adopted with the goal of promoting consistency and improving adherence.

Participants were provided diaries to record session compliance (session duration and intensity). Session compliance was monitored during weekly phone calls which were in addition to collecting information to help progress the NMES prescription, were used to maximise the compliance with the intervention, encourage continued increase in stimulation intensity where possible, and to identify and solve problems. In addition, due to the cognitive issues which can be experienced by individuals undergoing treatment such as memory loss^32^ written instruction manuals were provided with NMES units.

### Sample size calculation

A paired sample size calculation was conducted for the primary outcome of 30 s sit-to-stand using the formula:

$${\text{n}} \ge {\text{2 K}}{\upsigma }_{{\text{d}}}^{2} /\Delta ^{2}$$

K = 7.84; σ_d_ = Sd of the paired differences and Δ = Change to be detected^33^.

A standard deviation, 2.5 for within group change scores in the 30 s sit-to-stand performance was identified from pilot data from this study group (2 × case series) following a comparable, concurrent NMES exercise programme^14^. A mean change of 3 repetitions was sought, which is in line with the MCID reported in patients with comparator clinical populations^26,27^. With power set at 80% and alpha at 0.05 (two-tailed), a minimum of N > 12 participants was required for final analysis. Allowing for documented, high attrition rates (50%) in oncology trials^34^, N > 30 participants was targeted for recruitment.

### Data analysis

#### Parametric data

A Shapiro–Wilks test was carried out to test for normality. Data are presented as mean (SD) unless otherwise stated. A repeated measures Analysis of Variance (ANOVA) and Fisher LSD post hoc test assessed differences across the three time points (BL1 vs BL2, BL2 vs POST and BL1 vs POST) for 30STS and 6MWT performance. For the incremental session data, changes in maximum tolerable intensity, maximum heart rate (HRmax), RPE, and discomfort were compared using a paired T-test BL2 vs POST. Statistical significant was set a *p* < 0.05 for all parametric data.

#### Non-parametric data

A Friedman’s two-way ANOVA test was carried out to identify differences across the intervention for non-parametric data and non-normally distributed data. This included timed up an go (TUG), and QoL data. Post hoc analysis with Wilcoxon signed-rank tests was conducted with Fisher (LSD). All calculations were performed using SPSS, Version 24 software (SPSS Inc, Chicago IL, USA). Statistical significant was set a *p* < 0.05 for all non-parametric data.

### Ethical approval

This study was approved by the Health Council of Andalucía. All procedures performed in studies involving human participants were in accordance with the ethical standards of the institutional and/or national research committee and with the 1964 Helsinki declaration and its later amendments or comparable ethical standards.

## Results

**Figure 2:**
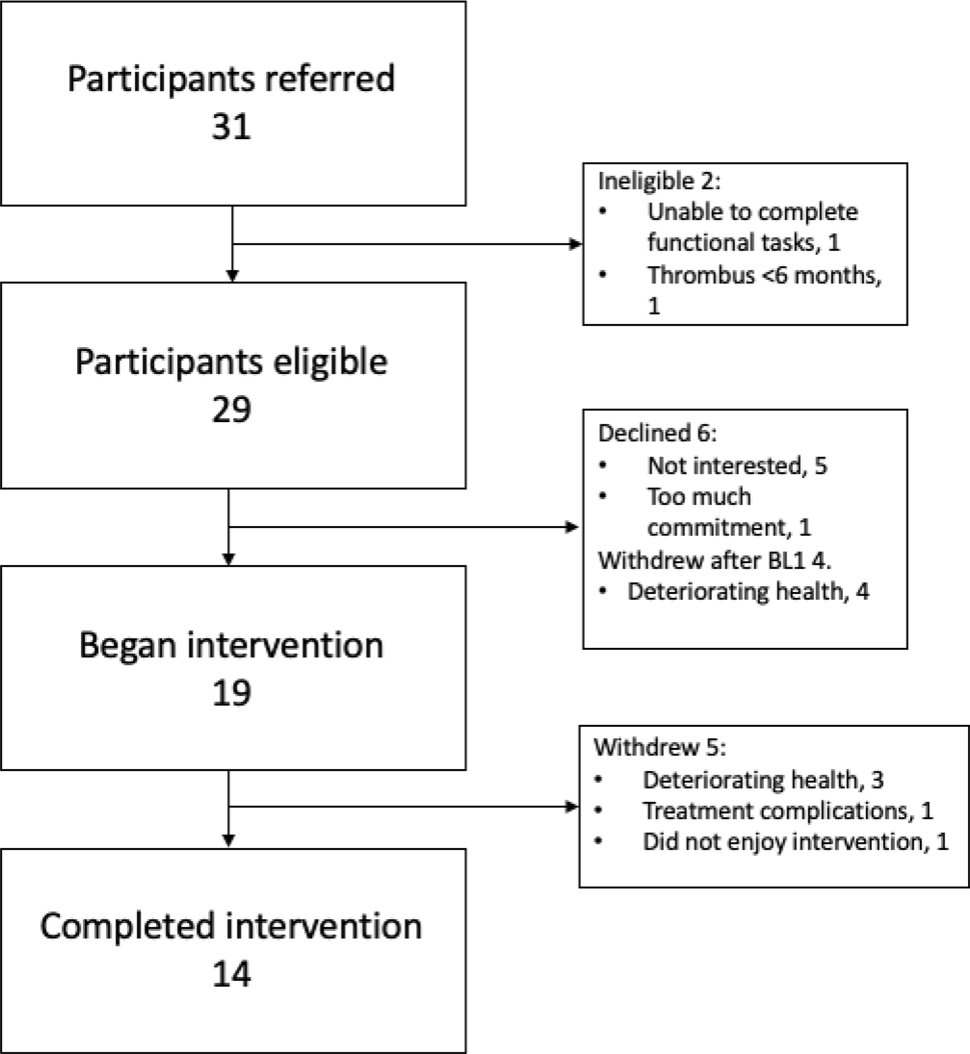
Study flow diagram.

Thirty-two study participants were identified over a 10-month period which commenced in February 2019. All participants were referred to participate by their treating oncologist at a private oncology clinic. Nine declined to participate with five stating lack of interest and four experiencing a deterioration in health. Twenty-three participants underwent BL1 assessment. Nineteen participants began the concurrent NMES exercise intervention. All four participants who withdrew after BL1 did so due to a deterioration in health. Completion of the study was achieved by 14 of the 19 (78%) participants who began the intervention (Fig. 2). No serious adverse events were reported. Four of the five participants who withdrew after beginning the intervention did so due to deterioration in their health, or complications associated with their treatment regime. One patient withdrew as they did not enjoy the local discomfort experienced during NMES sessions.

Study participants had a mean age of 62 years (SD 10). The baseline functional capacity of participants was low (mean 6MWT distance 308.1 m (168.3), and the majority of participants were documented as having poor functional status, ECOG 2 (61%) (Table 1).

**Table 1:** Baseline characteristics.

	No. of participants (n = 14)
**Demographic profile**	
Age (years)	
Mean (SD)	62 (10)
Range	48–75
Sex (m/f)	4/10
Married	8
**Medical profile**	
BMI (kg/m^2^)	
Mean (SD)	24.4 (6.7)
**Cancer diagnoses**	
Breast	4
Colon	1
Head and neck	1
Kidney	1
NSCLC	5
Rectal	1
Stomach	1
**ECOG status**	
ECOG 1	6
ECOG 2	8
**Cancer stage**	
III	3
IV	11
**Current treatment regimen**	
Radiotherapy	0
Chemotherapy	12
Hormone therapy	2
Targeted/immunotherapy	4

BMI; body mass index, ECOG; eastern cooperative oncology group.

Programme adherence data identify that for those who completed the intervention, the mean number of NMES exercise sessions completed was 11 (3) out a possible total of 14. The initial to final mean NMES exercise intensities reported by participants during the home-based intervention significantly increased from 56.7 mA (15.3) to 78.1 mA (29.5) for the LF-NMES phase (t(12) = -4.51, *p* = 0.001) and 48.7 mA (7.8) to 63.8 mA (29.7) for the HF-NMES phase (t(12) = −  3.23, *p* = 0.007).

**Figure 3:**
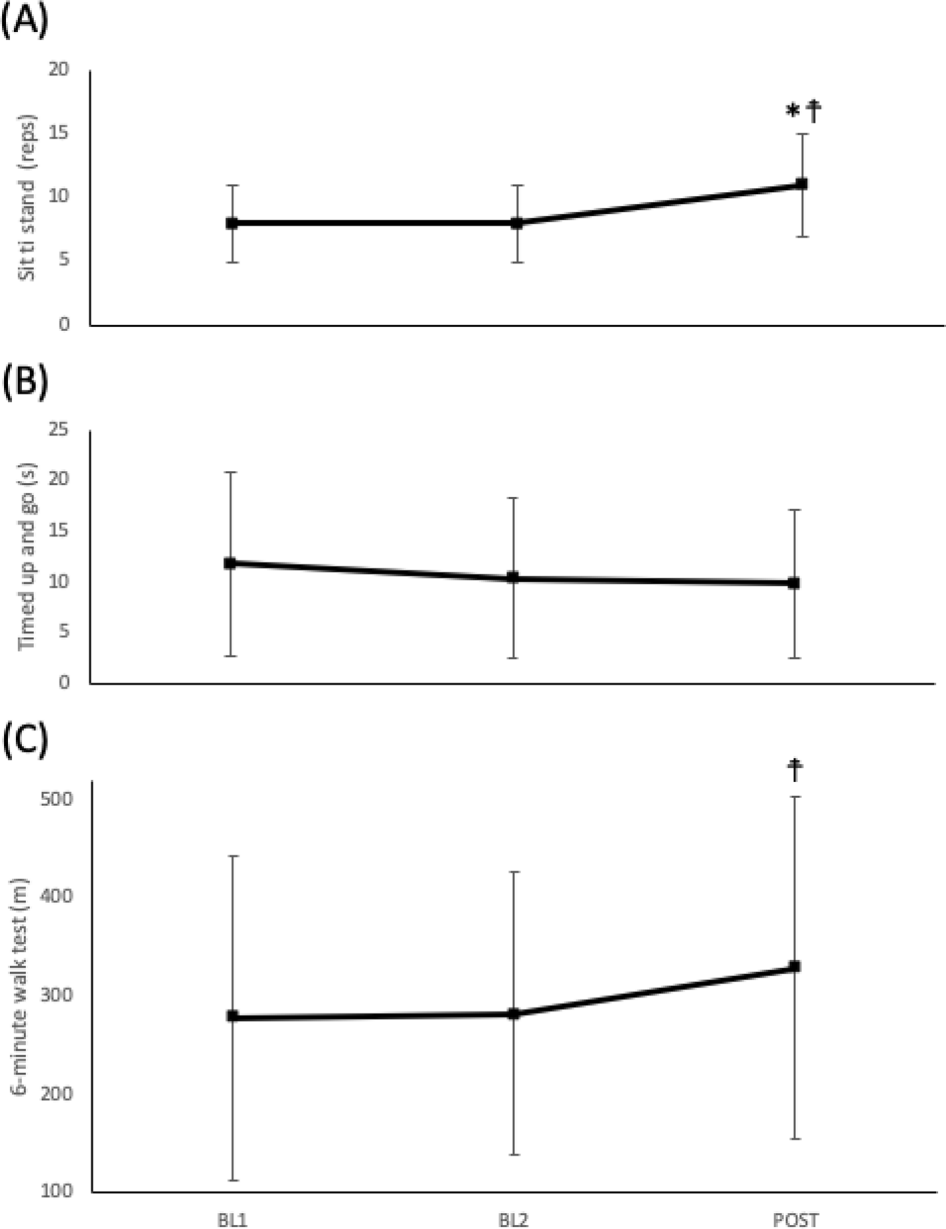
Group mean (SD) values across the study time period. Symbols indicate significantly different *p* < 0.05, vs BL1 (*) or BL2 (☨).

Mean results for the primary outcome measure, 30STS performance, and for secondary functional outcomes (6MWT, TUG) at each study phase (BL1, BL2 and POST) are summarised in Table 2. A repeated measures ANOVA showed that mean 30STS [F(1.111, 14.442) = 11.571, *p* = 0.003] and 6MWT (F(1.418, 18.434) = 5.385, *p* = 0.022] performance differed significantly between time-points. Post hoc tests using Fisher’s LSD revealed that STS performance improved by an average of 2.4 reps (2.8) (*p* = 0.007) and 6MWT performance improved by an average of 44.3 m (67.2) (*p* = 0.028) after the 4-week intervention (POST) from BL2 (Fig. 3). A Friedman test was carried out to compare the TUG performance over the intervention period. No significant differences were found across the intervention between time points $\chi 2$ (2) = 3.434, *p* = 0.180.

**Table 2:** Mean (SD) results for 30 s STS, TUG and 6MWT at BL1, BL2 and POST.

	BL1	BL2	POST	Within group comparison (*p* value)
30-s sit to stand (reps)	8 (3)	8 (3)	11 (4)	.003
Timed up and go (s)*	11.6 (7.6–16.5)	10.3 (6.7–14.3)	9.6 (6.7–14.0)	.180**
6-min walk distance (m)	304 (186)	308 (168)	352 (190)	.022

*Reported as median [interquartile range (IQR)]. **Friedman two-way ANOVA used as non-parametric alternative.

QoL data were not interval and were not normally distributed during normality tests. Group median results for all QoL functional scales and the fatigue symptom scale are presented at the three time points where differences across study time points were noted (Table 3).

**Table 3:** Group median (IQR) values and Friedman ANOVA results for quality of life function categories and fatigue symptom category.

	BL1	BL2	POST	Within-group comparison (*p* value)
Global QoL	50 (31–58)	50 (33–67)	38 (29–58)	$\upchi 2$ (2) = 2.178, * p* = .337
Physical	53 (20–67)	53 (33–75)	73 (55–80)	$\upchi 2$ (2) = 5.550, * p* = .062
Role	25 (0–67)	42 (0–67)	42 (13–75)	$\upchi 2$ (2) = 3.769, * p* = .152
Emotional	50 (25–67)	58 (31–75)	67 (50–75)	$\upchi 2$ (2) = 4.050, * p* = .132
Cognitive	58 (33–83)	67 (50–87)	74 (50–100)	$\upchi 2$ (2) = 5.214, * p* = .074
Social	33 (0–50)	42 (33–67)	33 (29–50)	$\upchi 2$ (2) = 2.263, * p* = .323
Fatigue	83 (53–100)	72 (39–89)	56 (33–72)	$\upchi 2$ (2) = 1.400, * p* = .497

Due to technical issues with the gas analyser no data from the POST period could be collected. As such mean values for peak NMES intensity, peak HR, RPE and discomfort are reported for the first and second incremental NMES sessions. During the second incremental session, peak NMES intensities achieved were significantly higher vs first incremental session (77.6 mA (28.6) vs 56.6 mA (14.6), *p* < 0.001). Peak HR and discomfort values were also higher at the second incremental session; however, these values were not significantly different (Table 4). There were no changes in peak RPE values reported at both incremental sessions.

**Table 4:** Comparison of group mean values for peak stimulation intensities, physiological response, RPE and discomfort during the first and second incremental NMES session.

	First incremental NMES session	Second incremental NMES session	*p* value
VO_2_ (ml min kg^−1^)	4.7 (1.1)	No data	N/A
Intensity (mA)	56.6 (14.6)	77.6 (28.6)	.000
Heart rate (beats min^−1^)	92.0 (21.0)	100.0 (18.9)	.107
Borg RPE (6–20)	10.6 (2.3)	10.8 (2.6)	.732
Discomfort (0–10)	6.6 (2.2)	7.2 (2.0)	.088

## Discussion

This is the first study to investigate the functional, physiological and subjective effects of a home-based concurrent NMES exercise intervention in participants with a cancer diagnosis, who were concurrently undergoing treatment and were deemed to have moderate or poor functional status. All participants had been identified by their oncologist to have limitations in exercise participation. The main finding of this study was that a concurrent NMES exercise intervention resulted in significant and clinically meaningful improvements in lower limb functional muscle strength and functional exercise capacity as measured by 30STS and 6MWT. Higher maximum LF-NMES intensities were achieved at a similar level of subjective discomfort during the second incremental NMES session suggesting early habituation to the intervention.

The primary reason for conducting this study was to assess the effects of a relatively modest dose of concurrent NMES exercise (2–5 × /week, 14 session over 4 weeks) on functional outcomes in participants with cancer. Previous work in oncology using this concurrent progressive approach has demonstrated a trend for improvement in lower limb strength and functional exercise capacity following NMES exercise application^14,15^; however, these studies were designed to provide preliminary data on safety and feasibility and the study design employed was uncontrolled in nature. Although randomised controlled trials (RCT’s) are the gold standard, these designs are not always pragmatic and may be undermined by issues including intervention delivery and participant recruitment and retention^35^. As such the current study used a two-week control period to address the difficulties described and the need for a no/sham NMES control group, with stability in this population across this control period. In addition to this, we attempted to control for residual treatment effects by timing assessments to treatment administration (at least 1-week post treatment infusion).

Although recent physical activity guidelines from the American College of Sports Medicine (ACSM) for individuals with cancer provide strong evidence for the participation in concurrent exercise (aerobic and strengthening) for improving physical function^8^, many patients undergoing active treatment experience exercise limiting complications. Exercise which uses concurrent NMES exercise has capacity to provide a pragmatic alternative to conventional exercise. In the current study, significant improvements in the primary outcome, 30STS performance were observed in favour of the concurrent NMES stimulation phase. Improvements in this outcome were reported in 9 of 14 participants, with eight participants achieving the MCID. Potential mechanisms behind muscle strength gains include greater motor unit recruitment which has been seen after 4 weeks of HF-NMES^36^; however, in the absence of supporting data this is speculative. It should be noted that although the improvement was significant, the increase was modest (3 reps, or 25%). In addition, when comparing 30STS change by functional group, no significant difference was found. These results are encouraging, meet the MCID reported in comparator clinical populations and highlights the potential use of concurrent NMES to elicit a strengthening effect in cancer patients unable to partake in conventional exercise.

Disabilities related to basic activities of daily living (ADL) such as walking are prevalent in > 50% of patients with cancer^20^. We observed significant improvements in 6MWT distance, with a mean increase of 44 m, (+ 14%) after the concurrent NMES exercise intervention. Improvements were reported in 11 of the 14 participants, with eight participants exceeding the MCID. Potential mechanisms behind these improvements include an increase in muscle oxidative capacity which is shown to occur following protracted exposure to LF-NMES^37^; however, again, without supporting data this is speculative. Of note, our results are greater than those reported by Banerjee et al.^13^, who noted a 7.5% (~ 39 m) increase in 6MWT distance following 6 weeks of LF-NMES in patients with chronic heart failure (CHF). Discrepancies between study results could be linked to the more overall deconditioned state of the participants in the current study (baseline 6MWT: 308 m vs 415 m). However, the additional stimulus of concurrent NMES exercise should also be considered. For example, reduced lower limb muscle strength is associated with impaired walking performance^38,39^. Therefore, the ability of concurrent NMES exercise to potentially target multiple body systems may well have provided greater benefits than either NMES exercise modality alone.

We observed no significant changes in any QoL categories. This is likely due to the study being underpowered to detect a change in these outcomes. However, it is also important to highlight that clinically meaningful changes in Global QoL, Physical QoL subscale and Fatigue symptom subscale were noted. Global QoL deteriorated by 12 points from BL2 to POST whilst both Physical QoL and Fatigue improved by 20 and 16 points respectively over the same time period. The deterioration in Global QoL is unsurprising despite clinically meaningful improvements in Physical and Fatigue subscales given that this health outcome is known to deteriorate across a patient’s treatment cycle, and encompasses a range of factors that may have a more varied response in individuals^8^. In addition, exercise interventions exceeding 12 weeks are recommended to see improvements in this health outcome^8^.

LF-NMES in healthy participants has been shown to elicit a comfortable and sustained therapeutic aerobic exercise response (> 50% VO_2max_), translating into an increase in aerobic capacity and exercise performance^12,21^. This training was also accompanied by a significant increase in maximum heart rate (HR_max_) at the second incremental NMES session^21^. We attempted to investigate the aerobic exercise response to LF-NMES in oncology. Unfortunately, complications with gas analyser equipment meant VO_2_ data was not possible. Therefore, we reported HRmax achieved during the first and second incremental NMES sessions. When compared with the HR_max_ response reported by Crognale et al.^21^, who targeted the same muscle groups in a healthy active group, HR_max_ achieved in the current study was lower at both the first [92.0 beats.min^−1^ (21.0) vs 114.1 beats.min^−1^ (20.7)] and second [100.0 beats.min^−1^ (18.9) vs 134.8 beats.min^−1^ (21.9)] incremental sessions. Differences may be explained by a lower absolute maximum NMES intensity [77.6 (28.6) mA vs 103.8 (18.0) mA], and leg fat free mass which is correlated with tolerance in more severe disease^40^.

In the absence of a central exercise response it is difficult to propose mechanisms behind aerobic exercise improvements following LF-NMES. However, improvements may have been mediated by peripheral muscle adaptations (increased oxidative capacity) to LF-NMES. Indeed, LF-NMES has been shown to enhance citrate synthase (CS) activity by 9 to 31% in healthy and patient populations when used `3x/week, for 4 to 10 weeks^37^. In patient populations such as those with COPD, the activity of CS has been correlated with the functional capacity of the individual^41^. Interestingly, treatments such as chemotherapy are associated with reduced mitochondrial density, mitochondrial dysfunction and reduced oxidative enzyme activity which can contribute to disrupted capacity for oxidative metabolism^42^. The observed disruption to mitochondrial dynamics can reduce the muscle’s ability to utilise effectively the oxygen that is delivered^43^. Therefore, despite no central physiological response, peripheral muscle adaptations following LF-NMES may help normalise oxidative enzyme activity towards a more oxidative phenotype and may contribute to improvements in functional exercise capacity. Future studies are warranted to establish the mechanisms behind adaptations to LF-NMES.

When identifying suitable outcome measures for future work in this population, the limitations of this cohort must be considered. Although the response to TUG was not significant, individuals with advanced cancer may struggle with prolonged periods of walking. As such, the 30STS or TUG tests may be the most appropriate outcome measures for focus in future work. An adapted Short Physical Performance Battery (SPPB), which includes the 30STS in favor of the 5STS (due to the floor effect) may be an appropriate battery of tests to assess the effectiveness of concurrent NMES exercise in cancer rehabilitation. Furthermore, working towards establishing a minimum data set for future work should consider the 30STS and TUG.

## Strengths and limitations

A strength of our study design was the inclusion of a control period in which we demonstrated stability in the participants included. In addition, we controlled for residual treatment complications by assessing participants at least 1-week pre/post treatment However, our study has several limitations. A parallel ‘standard care’ control group would have strengthened our results. Future work should consider an RCT design. Although we were successful in recruiting the minimum number of participants required to find significant change in our primary outcome, our sample size was still small and our study population is heterogeneous, likely leading to a lack of statistical power for some outcomes, and making it hard to draw definitive conclusions regarding the effects of concurrent NMES on functional and QoL outcomes. In addition, because of our small sample, we used Fisher LSD during our analysis when comparing groups across time periods. Fishers LSD is less conservative than Bonferroni correction, therefore increasing Type 1 error rate. As such, future studies with a larger sample are needed to confirm our results. Finally, we acknowledge that our sample was relatively young and may not be representative of those in an older and frailer oncology population.

## Conclusions

In conclusion, concurrent NMES exercise may be effective for enhancing functional muscle strength and exercise capacity in participants undergoing treatment for cancer. This study does not demonstrate that LF-NMES can elicit an aerobic exercise response in cancer patients. However, peripheral muscle adaptations following LF-NMES, combined with the muscle strengthening effects of HF-NMES may have contributed to functional improvements noted. Future work is required to elaborate on the mechanisms behind concurrent NMES exercise adaptations. A need remains to identify the most appropriate candidates for concurrent NMES exercise. Future large trials are required to expand on the findings of this study.
